# Modified Hodge test: A simple and effective test for detection of carbapenemase production

**Published:** 2011-12

**Authors:** A Amjad, IA Mirza, SA Abbasi, U Farwa, N Malik, F Zia

**Affiliations:** Department of Microbiology, Armed Forces Institute of Pathology, Rawalpindi Pakistan

**Keywords:** Modified Hodge test, disc diffusion, carbapenemases

## Abstract

**Background and Objectives:**

Resistance among bacterial isolates is the leading cause of increased mortality and morbidity worldwide. Carbapenems once thought to be effective are becoming ineffective mostly due to the emergence of carbapenemase. This study was designed to determine *in vitro* efficacy of Modified Hodge test for detection of carbapenemase production in Gram negative rods.

**Material and Methods:**

The study was done in the Department of Microbiology, Armed Forces Institute of Pathology Rawalpindi Pakistan from January 2010 to December 2010. A total of 200 Gram negative rods from different clinical samples were taken. Those isolates which showed intermediate or susceptible zones i.e 16mm-21mm on disc diffusion were included in the study. These isolates were then subjected to Modified Hodge test.

**Result:**

Out of 200 isolates, 138 (69%) were positive for carbapenemase production by Modified Hodge test. Out of 138 MHT positive organisms, the frequency of *E. coli* was 38%, followed by *Pseudomonas aeruginosa* (30%), *Klebsiella pneumoniae* (17%), *Acinetobacter baumannii* (12%)*, Citrobacter diversus* (2%) and *Enterobacter agglomerans* (1.4%).

**Conclusion:**

Modified Hodge test is a simple test which can be performed in the routine lab for detection of carbapenemases in isolates showing intermediate or sensitive zone diameter on disc diffusion.

## INTRODUCTION

Infections with resistant bacterial isolates are emerging as an important challenge in health care facilities. Antimicrobial resistance is associated with adverse outcomes, including increased mortality, hospital stay and costs. In addition, a delay in institution of effective therapy, inferior definitive therapy and greater virulence of some resistant strains are responsible for antimicrobial resistance (1, 2).

After methicillin resistant staphylococcus and extended spectrum beta lactamases, another beta lactamase causing resistance among Gram negative organisms is carbapenemase enzyme. This is an enzyme that hydrolyses a group of antibiotics called carbapenems (3, 4). Carbapenems are famously stable to AmpC β-lactamases and extended-spectrum-β-lactamases (5). This group is considered treatment of choice for infections caused by resistant strains of Gram negative bacteria. Unfortunately, resistance to carbapenems in enterobacteriacae is difficult to detect by routine disc diffusion method used by many microbiology laboratories.

Carbapenemases were formerly believed to be derived only from classes A, B, and D, but a class C carbapenemase (CMY) has been described. These enzymes fall into three of the Ambler classes of beta-lactamases A, B and D (6).

Detection of carbapenemases is difficult. It can be detected by phenotypic as well as genotypic methods (7). Among phenotypic tests, MHT is a relatively easy and simple test to be performed in a laboratory.

The study is being undertaken with a view to generally screen the Gram negative rods for the production of carbapenemase as these enzymes do not always produce resistant breakpoints for carbapenems using standardized susceptibility testing methods. The isolate may thus be reported as sensitive while still harboring carbapenemase enzyme resulting in potential treatment failure and dissemination of the resistant isolates.

## MATERIAL AND METHODS

This study was conducted in the Department of Microbiology, Armed Forces Institute of Pathology Rawalpindi Pakistan, from January 2010 to December 2010. A total of 200 Gram negative rods recovered from different clinical samples including pus, pus swabs, urine, tissue cultures and bronchoalveolar lavage were taken. The samples were cultured and the organisms isolated were identified by standard microbiological techniques. The antimicrobial susceptibility to carbapenems was done by disc diffusion method. Zone sizes were measured according to CLSI recommendations. The isolates which showed intermediate or susceptible zones for imipenem, i.e 16mm-21mm, were tested for carbapenemase production by Modified Hodge test, as CLSI recommends the MHT to be performed before reporting carbapenem susceptibility results if a clinical isolates has an elevated but susceptible carbapenem MIC (8).

A 0.5 McFarland dilution of the *Escherichia coli* ATCC 25922 in 5 ml of broth or saline was prepared. A 1:10 dilution was streaked as lawn on to a Mueller Hinton agar plate. A 10 µg meropenem or ertapenem susceptibility disk was placed in the center of the test area. Test organism was streaked in a straight line from the edge of the disk to the edge of the plate. The plate was incubated overnight at 35±2°C in ambient air for 16–24 hours.

Quality control of the carbapenem disks were performed according to CLSI guidelines. Quality control of the following organisms MHT Positive *Klebsiella pneumoniae* ATCC1705 and MHT Negative *Klebsiella pneumoniae* ATCC1706 were run with each batch of the test.

**Fig. 1:**
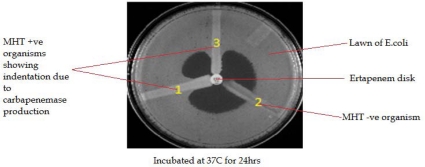
The MHT performed on a Muller Hinton Agar plate. (1) MHTpositive result (2) MHT negative result; and (3) a clinical isolate, positive result.

After 24 hrs, MHT Positive test showed a clover leaf-like indentation of the *Escherichia coli* 25922 growing along the test organism growth streak within the disk diffusion zone. MHT Negative test showed no growth of the *Escherichia coli* 25922 along the test organism growth streak within the disk diffusion as shown in Fig. 1.

## RESULTS

Out of a total 200 isolates which were showing intermediate or susceptible zone i.e16mm-21mm for imipenem, 138(69%) were positive for carbapenemase production by Modified Hodge test.

Carbapenemase producing organisms were E.coli 38%, followed by Pseudomonas aeruginosa 30%, Klebsiella pneumoniae 17%, Acinetobacter baumannii 12%, and Citrobacter diversus 2% and Enterobacter agglomerans were 1.4% (Table 1).

**Table 1:** Bacteria showing positive results in Modified Hodge test (n=138).

Sr. no.	Name of organism	Number of isolates
1	*Escherichia coli*	52 (38%)
2	*Pseudomonas aeruginosa*	42 (30%)
3	*Klebsiella pneumoniae*	23 (17%)
4	*Acinetobacter baumannii*	17 (12%)
5	*Citrobacter diversus*	3 (2%)
6	*Enterobacter agglomerans*	2 (1.4%)

## DISCUSSION

For more than 2 decades carbapenems have been considered the treatment of last resort for managing multidrug resistant infections caused by Enterobacteriacae. Carbapenemases, which hydrolyzes carbapenems and renders them inactive, have been increasingly reported in Asia and Europe, and more recently have been detected in Canada and the United States (10–14).

In 1996, the first isolate of *Klebsiella pneumoniae* Carbapenemase (KPC) producing organism was isolated in a clinical specimen from a hospital in North Carolina involved in the Intensive Care Antimicrobial Resistance Epidemiology (ICARE) surveillance program. KPCs were infrequently isolated until 2001, when KPC-producing Enterobacteriaceae were reported in several extended outbreaks in different hospitals of New York and New Jersey (15–17). KPCs have also been found in *K. oxytoca* (18), *Escherichia coli* (19), *Citrobacter* spp (20), *Pseudomonas aeruginosa* (21), *Salmonella* spp (22), *Serratia marcescens* (20), as well as *Proteus mirabilis* (23).

The Modified Hodge test as it is described today is a modified version of Hodge test which was used some years ago. Even the original Hodge test was evaluated utilizing the PCR confirmed IMP-1 and VIM-2 Metallo β-lactamase (MBL) producing isolates. The original Hodge test utilized imipenem 10µg disc which gave fairly good results as it detected 67% cases of MBL producing *Pseudomonas aeruginosa* and *Acinetobacter* spp. The other 13 cases which gave initially equivocal or negative results were retested and were declared positive using imipenem discs containing zinc sulphate (24).

In another study done at Centers for Disease Control and Prevention in Atlanta GA in 2007, 45 isolates (26 of *K. pneumoniae*, 9 of *K. oxytoca*, and 10 of *E. coli*) were evaluated by Modified Hodge test and all of them were validated by PCR for the detection of KPC activity with 100% sensitivity and specificity (25). This shows that Modified Hodge test is a very sensitive and reliable test for detection of carbapenemases.

A study was carried out in Greece in 2007 to evaluate different laboratory tests for detection of MBLs in Enterobacteriacae. Modified Hodge test detected 98% cases keeping PCR as the gold standard while only 0.03% was detected as false positive (26).

Recently a study done in Pennsylvania revealed that out of 85 ertapenem intermediate or resistant isolates, 75% were KPC (*Klebsiella pneumoniae* carbapenemase) positive tested by Modified Hodge test and further confirmed by PCR (27). While in our study out of 100 isolates which were carbapenem intermediate or sensitive, 69% showed the presence of carbapenemase by Modified Hodge test only.

Recently a latest version of carbapenemases, New Delhi metallo-β-lactamase-1(NDM-1) is making headlines all over the world (28). Though the definite diagnosis of NDM-1 rests on PCR but Modified Hodge test can be a very useful screening test for suspecting such cases for epidemiological purpose.

In many healthcare facilities around the world, bacterial pathogens that express multiple resistance mechanisms are becoming rampant, complicating treatment and increasing both human morbidity and financial costs. This necessitates the need for detecting the resistant bacteria so that unnecessary use of broad spectrum antimicrobials can be avoided. The significant finding of our study was the fact that quite a large percentage i.e 69% of our isolates which showed intermediate or susceptible zone sizes on disc diffusion were detected positive by MHT indicating the huge importance of this simple test. Hence majority of such patients would be prescribed carbapenems which would be disastrous on two accounts, firstly the patient would end up in treatment failure and secondly unnecessary usage of carbapenems would further expose this antimicrobial with potential for more resistance.

In conclusion, Modified Hodge test is an easy and simple test to be performed to detect carbapenemases producing bacteria. There is a very high percentage of carbapenemases producing Gram negative rods in our setup. It is imperative that all isolates showing intermediate or sensitive zone diameter on disc diffusion be tested for production of carbapenemases by Modified Hodge test to avoid treatment failures and development of resistance due to unnecessary use of this class of antibiotic.

## References

[1] Centers for Disease Control and Prevention (2009). Guidance for control of infections with carbapenem-resistant or carbapenemase-producing enterobacteriacae in acute care facilities. MMWR.

[2] CLSI Guidance for control of infections with carbapenem-resistant or carbapenemase producing Enterobacteriaceae in acute care facilities CLSI home.

[3] Schwaber M, Carmeli Y (2006). Antimicrobial resistance and patient outcomes: The hazards of adjustment. Crit care.

[4] Gary D O, Margret FC (2010). Carbapenemases: A brief review for pediatric infectious disease specialists. Pediatr Infect Dis J.

[5] Swenson M J, Hindler F J, Jorgensen H J, Baron E J, Jorgensen H J, Landry M L, Pfaller M A (2007). Special Phenotypic Methods for Detecting Antibacterial Resistance. Manual of Clinical Microbiology.

[6] Carbapenemases Beta-lactamase-Wikipedia, the free encyclopedia. http;//en.wikipedia.org/wiki/Beta-lactamase.

[7] Tenover C F (2006). Mechanisms of Antimicrobial Resistance in Bacteria. AM J Med.

[8] Clinical and Laboratory Standards Institute CLSI (2010). Screening and Confirmatory Tests for Suspected Carbapenemases Production.

[9] Centers for disease control and prevention Modified Hodge test for carbapenemase detection in enterobacteriaceae. http://www.ndhealth.gov/microlab/Uploads/HodgeTest.pdf.

[10] Chu Y W, Afzal-Shah M, Houang E T S, Palepou M F I, Lyon D J, Woodford N (2001). IMP-4, a novel metallo-β-lactamase from nosocomial Acinetobacter spp. collected in Hong Kong between 1994 and 1998. Antimicrob Agents Chemother.

[11] Iyobe S, Kusadokoro H, Ozaki J, Matsumura N, Minami S, Haruta S (2000). Amino acid substitution in a variant of IMP-1 metallo-β-lactamase. Antimicrob Agents Chemother.

[12] Lee K, Lim JB, Yum JH, Yong D, Chong Y, Kim JM (2002). *bla*
_VIM-2_ cassette-containing novel integrons in metallo-β-lactamase-producing *Pseudomonas aeruginosa and Pseudomonas putida* isolates disseminated in a Korean hospital. Antimicrob Agents Chemother.

[13] Migliavacca R, Docquier JD, Mugnaioli C, Amicosante G, Daturi R, Lee K (2002). Simple microdilution test for detection of metallo-β-lactamase production in Pseudomonas aeruginosa. J Clin Microbiol.

[14] Tolman MA, Rolston K, Jones RN, Walsh TR (2002). Molecular characterization of VIM-4, a novel metallo-beta-lactamase isolated from Texas: report from the cancer surveillance program (2001).

[15] Bratu S, Brooks S, Burney S, Kochar S, Gupta J, Landman D (2007). Detection and spread of *Escherichia coli* possessing the plasmid-borne carbapenemase KPC-2 in Brooklyn, New York. Clin Infect Dis.

[16] Lomaestro BM, Tobin EH, Shang W, Gootz T (2006). The spread of *Klebsiella pneumoniae* carbapenemase- producing K. pneumoniae to upstate New York. Clin Infect Dis.

[17] Bratu S, Mooty M, Nichani S, Landman D, Gullans C, Pettinato B (2005). Emergence of KPC-possessing *Klebsiella pneumoniae* in Brooklyn, New York: epidemiology and recommendations for detection. Antimicrob Agents Chemother.

[18] Yigit H, Queenan A M, Rasheed K J, Biddle W J, Domenech-Sanchez A, Alberti S (2003). Carbapenem- resistant strain of Klebsiella oxytoca harboring carbapenem- hydrolyzing β-lactamase KPC-2. Antimicrob Agents Chemother.

[19] Navon-Venezia S, Chmelnitsky I, Leavitt A, Schwaber M J, Schwartz D, Carmeli Y (2006). Plasmid-mediated imipenem- hydrolyzing enzyme KPC-2 among multiple carbapenem- resistant *Escherichia coli* clone in Israel. Antimicrob Agents Chemother.

[20] Deshpande L M, Rhomberg P R, Sader H S, Jones R N (2006). Emergence of serine carbapenemases (KPC and SME) among clinical strains of Enterobacteriaceae isolated in the United States medical centers: report from the MYSTIC program (1999–2005). Diagn Microbiol Infect Dis.

[21] Villegas MV, Lolans K, Correa A, Kattan JN, Lopez JA, Quinn JP (2007). First identification of *Pseudomonas aeruginosa* isolates producing a KPC type carbapenem- hydrolyzing β-lactamase. Antimicrob Agents Chemother.

[22] Miriagou V, Tzouvelekis LS, Rossiter S, Tzelepi E, Angulo FJ, Whichard JM (2003). Imipenem resistance in a *Salmonella* clinical strain due to plasmid-mediated class A carbapenemase KPC-2. Antimicrob Agents Chemother.

[23] Tibbetts R, Frye JG, Marschall J, Warren D, Dunne W (2008). Detection of KPC-2 in a clinical isolate of *Proteus mirabilis* and first reported description of carbapenemase resistance caused by a KPC β-lactamase in P. mirabilis. J Clin Microbiol.

[24] Lee K, Lim YS, Yong D, Yum JH, Chong Y (2003). Evaluation of the Hodge test and the imipenem-EDTA double disk synergy test for differentiating metallo-β- lactamase-producing isolates of *Pseudomonas* spp and *Acinetobacter* spp. J Clin Microbiol.

[25] Anderson KF, Lonsway RD, Rasheed KJ, Biddle J, Jensen B, McDougal LK (2007). Evaluation of methods to identify the *Klebsiella pneumoniae* carbapenemase in Enterobacteriaceae. J Clin Microbiol.

[26] Galani I, Rekatsina DP, Hatzaki D, Plachouras D, Souli M, Giamarellou H (2008). Evaluation of different laboratory tests for the detection of metallo-β-lactamase production in Enterobacteriaceae. J Antimicrob Chemother.

[27] McGettigan SE, Andreacchio K, Edelstein PH (2009). Specificity of Ertapenem Susceptibility Screening for Detection of *Klebsiella pneumoniae* Carbapenemases. J Clin Microbiol.

[28] Kumarasamy KK, Toleman AM, Walsh RT, Bagaria J, Butt F, Balakrishnan R (2010). Emergence of a new antibiotic resistance mechanism in India, Pakistan, and the UK: a molecular, biological, and epidemiological study. Lancet Infect Dis.

